# Advanced Analytical Approach Based on Combination of FT-IR and Chemometrics for Quality Control of Pharmaceutical Preparations

**DOI:** 10.3390/ph15060763

**Published:** 2022-06-18

**Authors:** Martina Foschi, Mariagrazia Marziale, Alessandra Biancolillo

**Affiliations:** Department of Physical and Chemical Sciences, University of L’Aquila, Via Vetoio, Coppito, 67100 L’Aquila, Italy; martina.foschi@univaq.it (M.F.); mariagrazia.marziale@student.univaq.it (M.M.)

**Keywords:** pharmaceutical drug authentication, classification, expired drugs, ATR-FTIR, discriminant analysis, SPORT-LDA, ensemble preprocessing, ASCA, DOE

## Abstract

Background: The present work represents a feasibility study for the realization of an analytical method finalized to the detection of expired antibiotic tablets. The work focuses on a specific antibiotic drug and represents the preliminary study upstream of a larger-scale work. Methods: attenuated Total reflection-Fourier transform infrared spectroscopy (ATR-FTIR) spectra coupled with sequential preprocessing through an orthogonalization (SPORT) chemometric approach were used to discriminate between expired and compliant tablets. Conclusions: The highest predictive accuracy (93.3% of correct classification rate in external validation, corresponding to 1 misclassified test sample over 15) was achieved by analyzing intact tablets. This represents an excellent result because it gives indications regarding the possibility of determining, in a completely non-destructive way, the presence of expired drugs.

## 1. Introduction

The quality control of pharmaceutical drugs is a serious concern for the World Health Organization (WHO) because, without the guarantee that these products meet the required standards of quality, safety, and efficacy, any health service is compromised [1]. According to the Food and Drug Administration (FDA), pharmaceutical companies must ensure the full efficiency and safety of the marketed product throughout the established shelf-life, when it is stored in the original unopened container and under recommended conditions [2]. Consequently, drug quality and stability control are among the main issues in pharmaceuticals [3,4]. The quality of drug products is not only a function of time and storage conditions but is closely linked to the manufacturing process, including appropriate packaging and formulation [5]. Indeed, excipients and tablet coatings play a key role in protecting active pharmaceutical ingredients (APIs) from environmental factors, such as light and oxygen exposure, humidity, and temperature [6,7]. Hence, stability tests are mandatory to determine the expiration date but are also helpful to provide valuable information on how to handle and store drug products and to guide formulation stabilization strategies.

Despite always being recommended to not use expired drugs, some types of medicine deserve more attention than others. For instance, antibiotics are one of these because, over time, they can develop by-products, leading to hepatotoxicity or adverse reactions [8]. However, the non-conscientious use of these drugs is more common than generally perceived, causing drug wastage and potential therapeutic failures in the future [9,10]. A study conducted in Palestine confirmed that many people are used to storing different types of antibiotics at home for future self-medication, but in several cases, they were found to be expired or with no clear expiry date [11,12]. It has to be pointed out that studies on the stability and prescription of drugs, in relation to the expiration date, have, as a necessary condition, the storage of drugs in optimal conditions.

Orero et al. concluded that keeping antibiotics in a domestic environment is a major factor that increases cost, reduces efficiency, and decreases the quality of antibiotic treatment at the community level [13]. The study demonstrated that a survey of stored antibacterial agents is useful in drawing attention to drug wastage, which is a serious issue, since it has an economical and ethical cost [14].

Starting from these considerations, the present work has, as one of its goals, to serve as a feasibility study for developing a rapid, non-destructive, and cost-effective detection tool for an expired antibiotic. The pharmaceutical drug under investigation is commercialized as Augmentin (or augmentine or augmentin duo) in several European countries and the USA. It is a combination of two different APIs: amoxicillin trihydrate and clavulanate potassium salt in a 7:1 ratio. To clavulanate, which works as a suicide inhibitor of beta-lactamase enzymes, can be ascribed the main side effects and it is, by far, a less stable component in the formulation, representing the overall stability-limiting factor [15].

In the literature, there are several published studies aimed at assessing the stability of different amoxicillin/clavulanate formulations; most of them comply with the United States Pharmacopeia (USP) and the International Council for Harmonisation (ICH) guidelines, in which the validation of quantitative high-performance liquid chromatography (HPLC) methods are foreseen for the quality control of drug products [16,17,18,19]. Du and co-workers characterized the degradation profiles of different medications after being stored on the international space station for several months. Determining APIs by ultra- and high-performance liquid chromatography and classifying the medications according to the (USP) criteria, they found that only in the case of clavulanate, the rate of degradation was faster on the ground than in space. This evidence was probably due to the susceptibility of the compound to uncontrolled relative humidity [20].

Dobrova et al. transferred and validated the HPLC method for quality determination of amoxicillin/clavulanate tablets to an eco-friendlier UPLC approach [21]. However, all the proposed methods are destructive, require long sample preprocessing, and are not completely environmentally friendly. Several analytical approaches were developed and validated by employing both UV and FT-IR spectroscopies to quantify APIs in different formulations, demonstrating the various applicative advantages of the spectroscopic-based methods [22,23,24].

As far as we currently know, only a single study relied on chemometrics to exploit untargeted spectroscopic profiles for a quantitative non-destructive analytical method. Based on NIR spectroscopy and partial least squares regression, the approach was fully validated according to the ICH guidelines to quantify tablet water content and amoxicillin dimer from this API degradation [25].

In the present work, chemometrics was employed to take advantage of the ATR-FTIR spectra information in all Augmentin tablets and their corresponding powder. The design of experiment and ASCA [26] were used to determine the instrumental settings producing the most intense spectroscopic signal in the case of ground tablets. Eventually, the performances of the models were compared to assess whether there was a real need to ground the medication and evaluated to confirm the feasibility of a simple, qualitative, cheap, and potentially non-destructive method able to differentiate between expired and compliant Augmentin tablets.

## 2. Results and Discussion

**Figure 1:**
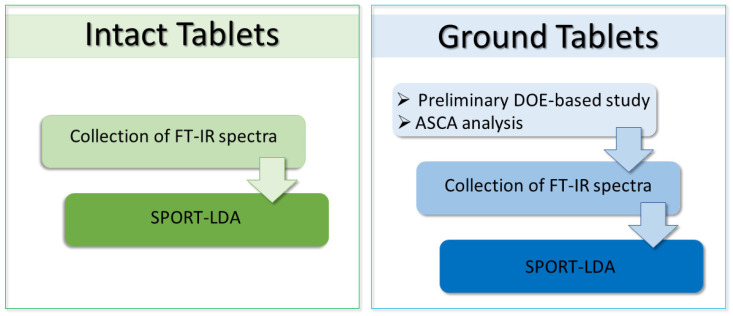
Chemometric workflow.

Due to the different physical nature of the analyzed samples (i.e., intact or ground tablets), two slightly diverse analytical workflows were applied (schematized in Figure 1). At first, spectra were collected on tablets as they were, and then they were preprocessed and classified by SPORT-LDA. On the other hand, since the spectra collected on ground tablets were noisier than those on the entire specimens, the procedure applied to these samples had an additional preliminary DOE-based study for the optimization of the instrumental parameters. In particular, a full-factorial design was used for investigating the effect given by the instrumental variation in the ATR module pressure, the spectral resolution, and the number of scans (more details on the DOE are given in Section 2.1) on the signals. After the collection of spectra, finalized for the preliminary investigation, ASCA was used to determine what spectroscopic conditions would improve the overall intensity.

Once ASCA was calculated, scores and loadings of the significant effects were investigated in order to detect what factor level would provide the highest signal intensity. Significancy was assessed by permutation tests (1000 permutations).

Eventually, ground samples of expired and compliant Augmentin tablets were classified by SPORT-LDA.

Although two different initial procedures were followed for samples of diverse natures, some common chemometric/statistical precautions were taken into consideration in both elaborations.

Specifically, in both cases, 36 signals, associated with 36 samples (11 expired and 25 compliant), were available; these were divided into a training and a test set, in order to enable external validation of the calibration models. The training set contained 21 spectra, 6 associated with expired samples and 15 with the compliant ones; on the other hand, the test set, made of 15 signals, included 5 expired and 10 compliant objects.

Calculating SPORT-LDA models, the same pre-processes were tested: standard normal variate (SNV), first derivative (D1), and second derivative (D2) (Savitzky–Golay, 15 point window, second-order polynomial for D1, and third-order polynomial for D2). The order of the input blocks was always the same, i.e.: raw spectra as the first input block, ($X_{1}$), data preprocessed by SNV as the second predictor ($X_{2}$), spectra pretreated by D1 as the third one ($X_{3}$), and signal preprocessed by D2 as the fourth block ($X_{4}$).

In all models, the optimal number of LVs to be extracted from each block was defined by means of a five-fold cross-validation procedure, testing all the possible combinations of LVs (for all blocks) between 0 and 8. A 0 value as the number of LVs means the related block was discarded by the model.

Calculations were run in Matlab (v.9.3, R2015b; The Mathworks, Natick, MA, USA) using in-house routines.

### 2.1. Analysis of Intact Tablets

Spectra were imported in Matlab and organized as described at the beginning of Section 3. 

**Figure 2:**
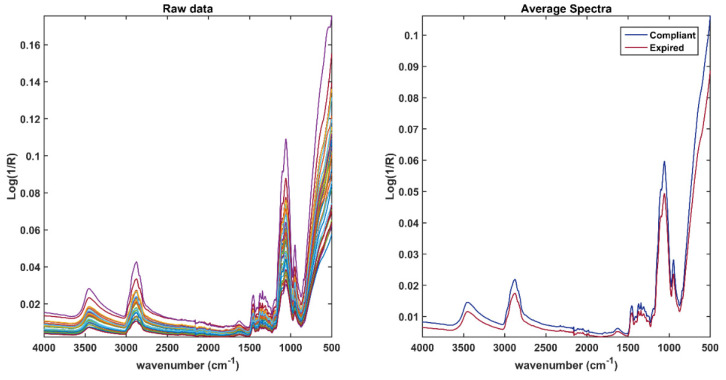
Raw spectra collected on the entire tablet (**left** plot); average spectra of expired (red) and compliant tablets (**right** plot).

All the available spectra are shown in the left plot of Figure 2, whereas, in the right plot, the average spectra per class are displayed. As expected, the spectra collected show a strong contribution from the excipients, in particular, from those used for the outer coating of the tablet. Signals attributable to HydroxyPropyl MethylCellulose (HPMC), which is a common excipient used as a pharmaceutical film, predominate on the spectrum. An HPMC polymer is often implemented as a tablet-coating, as in the case of those analyzed in this study. In the spectra, the signal at 1338 cm^−1^ that could be caused both by the hydroxyl group bending and the vibrational mode of titanium dioxide is well distinguishable [27]. This latter is used in this formulation as an additional coating and pigment. A wide band between 3500 cm^−1^ and 3300 cm^−1^, given by the O–H stretching (present in all the excipients and the APIs) is observable [28,29], whereas the peak at approximately 2877 cm^−1^ is attributable to the symmetrical and asymmetrical stretching of –C–H. In the fingerprint region, approximately between 1500 cm^−1^ and 800 cm^−1^, the main characteristic bands of carbohydrates are visible, in particular, peaks at 1135 cm^−1^ and 986 cm^−1^, which are due to the vibration of glycosidic bonds and of the C–O elongation in the C–O–C glycosidic bond [30]. Although the predominant signals are attributable to the presence of excipients (in addition to those already mentioned, there are other inactive ingredients such as colloidal silicon dioxide, magnesium stearate, microcrystalline cellulose, polyethylene glycol, and sodium starch glycolate), it is plausible that the spectra of the active ingredients, although not very recognizable, are present in the spectroscopic profile of the entire tablet (more details on these will be given in Section 2.2, discussing the spectra of ground tablets).

#### SPORT-LDA Analysis

All the spectra collected for the development of the classification tool were imported into Matlab and organized as described at the beginning of Section 3.

**Figure 3:**
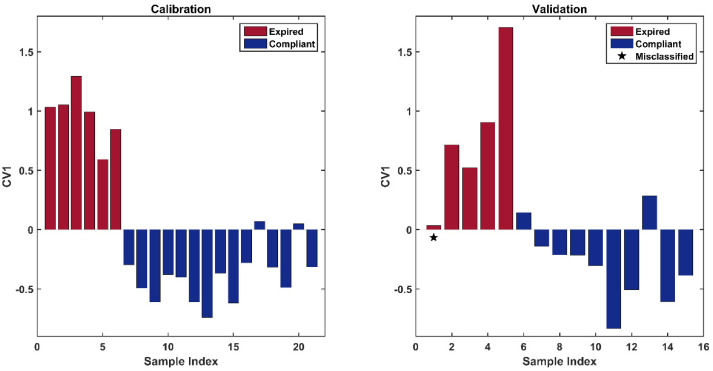
Canonical variate scores of calibration samples (**left** plot); canonical variate scores of validation samples (**right** plot). The black star indicates the misclassified sample.

The optimal number of LVs to be extracted was 1, 2, 5, and 0 for $X_{1}$, $X_{2},\;X_{3}$, and $X_{4}\;$, respectively, indicating the raw data and the blocks preprocessed by SNV and the first derivative brought relevant information to the model, whereas the matrix pretreated by the second derivative was discarded. The resulting calibration model provided a cross-validated global accuracy of 80.9%, corresponding to correct classification rates (in CV on the training set) of 100.0% and 80.0%, for class compliant and expired, respectively. The application of the model to the validation set led to the correct classification of all the compliant individuals and to the misclassification of only one expired sample, corresponding to a total accuracy (in prediction) of 93.3%. Results can also be appreciated in Figure 3, where the canonical variate scores for calibration (left plot) and validation (right plot) are shown.

The figure reveals a clear trend among samples; in particular, expired drugs present highly positive CV scores, whereas the compliant drugs have negative or slightly positive values for CV1. The misclassified test sample (highlighted by a black star in the right plot in Figure 3) is an expired individual that does not respect this trend and presents a CV1 value close to zero.

**Figure 4:**
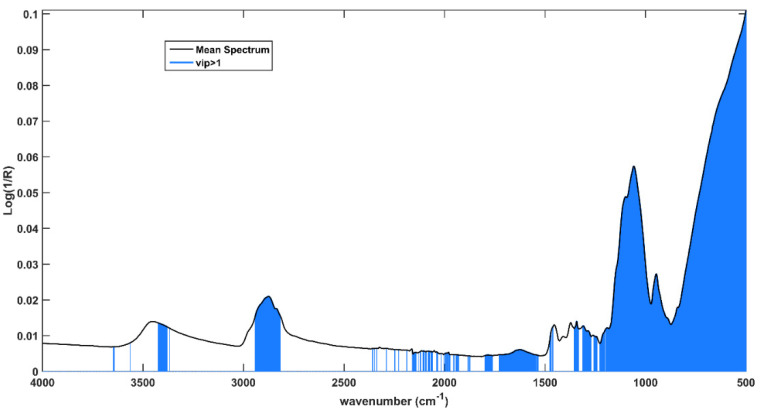
Average spectrum collected on the intact tablets. Spectral variables presenting VIP > 1 are highlighted in light blue.

Variable importance in projection (VIP) analysis was used to individuate which spectral variables are the most relevant for the solution of the classification problem. This approach allows the calculation of VIP indices, which provide an indication of the contribution of every single variable to the model. Customarily, variables presenting a VIP index higher than 1 are considered relevant. In Figure 4, the solid black line represents the mean spectrum and variables associated to VIP indices >1 are highlighted in sapphire blue.

It is evident that the fingerprint region is the most relevant spectral range; in fact, almost all variables between 1200 cm^−1^ and 500 cm^−1^ present VIP > 1, and many of them result as relevant in all the input blocks. Moreover, the contribution associated to the variables belonging to the peak centered at 2875 cm^−1^ and at (approximately) 3400 cm^−1^, ascribable to the symmetrical and asymmetrical stretching of -C-H and of O-H, appears relevant. Eventually, the last block is the only one presenting VIP > 1 in a range of 2400–1600 cm^−1^, where weak signals can be found at 1640 cm^−1^ and 1740 cm^−1^, which can be associated with the water content and the stretching of the carbonyl groups, respectively. This evidence probably indicates different humidity degrees and oxidation states between expired and compliant tablets.

### 2.2. Analysis of Ground Tablets

**Figure 5:**
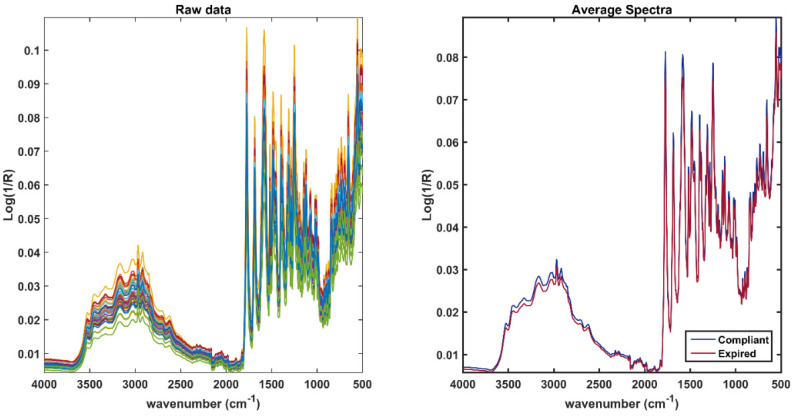
Raw spectra collected on the ground tablet (**left** plot); average spectra of expired (red) and compliant tablets (**right** plot).

By comparing the collected spectra (Figure 5) with those reported in the literature, some peaks of the two active ingredients can be recognized, in particular:

The band in the region between 3200 cm^−1^ and 3500 cm^−1^ is associable to the O-H stretching of the phenol of Amoxicillin and of the carboxylic group present in both Amoxicillin and clavulanate [24]. Within the same range also, the N-H stretching of the primary amine and the secondary amide of Amoxicillin fall. The peaks around 3000 cm^−1^ are attributable to the symmetrical and asymmetrical stretching of –C–H. One of the most characteristic bands of both active ingredients is between 1700 and 1800 cm^−1^, indicating the stretching of the carbonyl (C=O) of the β-lactam ring. Peaks between 1656 cm^−1^ and 1720 cm^−1^ are associable to the bending of the primary amine N-H_2_ of Amoxicillin, whereas the peak at about 1650 cm^−1^ is attributable to the stretching of C=O of the secondary amide. Eventually, the peaks in the region between 1500 cm^−1^ and 1300 cm^−1^ are associable to the C=C stretching of the aromatic ring and the N–H bending of the secondary amide, whereas the peak at 1250 is provided by the C–N stretching of the primary amine [30].

#### 2.2.1. ASCA

As anticipated, 37 spectra were collected according to the DOE described in Section 3.1 and analyzed by ASCA.

ASCA indicated only pressure as a significant factor (corresponding to *p*-value < 0.001).

**Figure 6:**
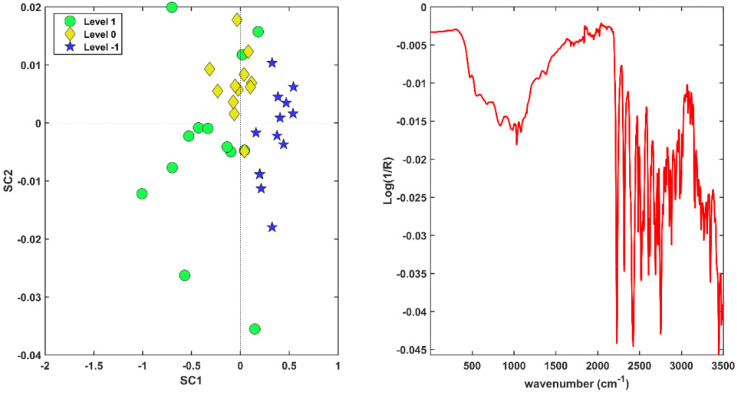
Scores (**left**) and loadings plot (**right**) associated to the ASCA model on effect pressure. Legend: pressure level 1: green dots; pressure level 0: yellow diamonds; pressure level -1: blue stars.

In particular, by looking at the loadings along SC1 (Figure 6), it is clear that the variation in the factor results in an overall change in intensity in the spectral profile. Observation of the score plot reveals that the maximum intensity corresponds to a negative score on SC1. The trials marketed as level 0, which correspond to a middle pressure of 50, generate a zero-centered cluster. The highest level (80) of the factor produced a distribution mainly localized at negative scores of SC1. Consequently, the spectral dataset collected with the aim of discerning compliant and expired samples was acquired, taking care of using a pressure of 80. As the other two factors (resolution and scans) were not significant, it was decided to reduce the spectrum noise by choosing a resolution of 8 cm^−1^ and the highest level of scans (24).

#### 2.2.2. SPORT-LDA Analysis

The same procedure described for intact tablets was followed for the ground ones. The order of the input block was the same as pointed out previously. In this case, the optimal number of LVs to be extracted from the different blocks was 1, 2, 0, and 0, for predictors $X_{1}$, $X_{2},\;X_{3}$, and $X_{4}\;$, respectively, indicating only the raw block and the one preprocessed by SNV contribute to the model.

**Figure 7:**
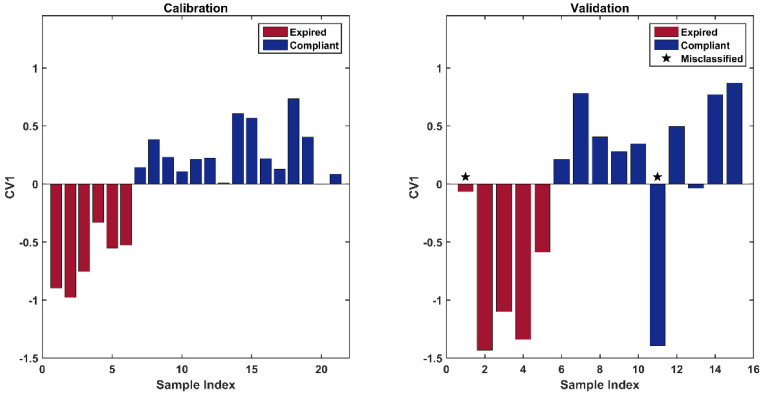
Canonical variate scores of calibration samples (**left** plot); canonical variate scores of validation samples (**right** plot). The black star indicates the misclassified sample.

This calibration model led to a cross-validated accuracy of 100.0% (on training samples) and highlighted an opposite trend with respect to the previous analysis. In fact, as it can be seen in Figure 7, in this case, expired samples present negative values of CV1, whereas the compliant individuals have positive ones. The application of the classification model to the test set led to the misclassification of only one sample per class, leading to a total correct classification rate of 86.7% in the validation set. This corresponds to a correct classification rate of 80.0% and 90.0% on expired and compliant samples, respectively.

**Figure 8:**
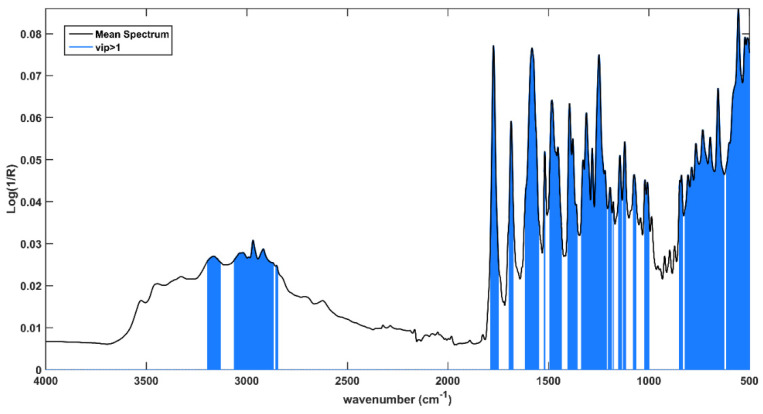
Average spectrum collected on the ground tablets. Spectral variables presenting vip > 1 are highlighted in light blue.

Further, in this case, VIP analysis was used to investigate the most meaningful variables for the determination of the expired tablets (Figure 8). This made apparent that the wide band around 3000 cm^−1^ (associable to O-H stretching present in all the excipients and the APIs) and peaks between 1772 cm^−1^ and 500 cm^−1^, ascribable to different compounds in samples (more details are reported above at the beginning of Section 2.1), contribute to the discrimination of expired and compliant samples.

## 3. Materials and Methods

### 3.1. DoE Plan and Samples

The design of experiment was taken into account to rationalize preliminary experiments and obtain a higher quality of information with a minimum number of trials. In this context, three instrumental parameters were evaluated, i.e., the pressure (expressed as a percentage of the maximum allowed force to adhere the sample onto the crystal), the spectral resolution, and the number of scans. According to a three-level full factorial design, all the possible combinations of the experimental variables were tested on a single ground tablet, obtaining 27 experiments on a symmetrical experimental domain. The three variables mentioned above were tested at three different levels, which will be indicated with −1, 0, and 1 and correspond to: 20–50–80 for the ATR module pressure; 2 cm^−1^, 4 cm^−1^, and 8 cm^−1^ for the resolution; and 8, 16, and 24 for the number of scans. Given the high spurious variability in the spectroscopic technique, which is greater when working with powders, several replicates were performed at the center and at each vertex of the experimental domain, obtaining a final number of 37 measurements. The obtained experimental matrix was exported and processed by applying ASCA. After identifying the instrumental working conditions, it was possible to continue the analysis of the samples. All the batches were kept at room temperature (reproducing common in-house storage). Three different lots were analyzed and, of those, only one was found to be expired at the time the analyses were carried out. All the lots were produced by Glaxosmithkline (Brentford, UK) and sold as Augmentin (Ph.Eur).

### 3.2. Collection of IR-Spectra 

Spectra were collected exploiting an FT-IR spectrometer (PerkinElmer Spectrum Two™-PerkinElmer, Waltham, MA, USA) equipped with a PerkinElmer Universal Attenuated Total Reflectance (uATR) diamond crystal (single bounce). The investigated spectral range was from 4000 cm^−1^ to 400 cm^−1^. Spectra on the entire tablets were acquired with a 4 cm^−1^ resolution, collecting 24 scans. Collecting ground samples, the same figures of merit were dictated by the above-mentioned DOE, and corresponded to 8 cm^−1^ and 24 scans, respectively. Between one measure and another, the crystal was cleaned with methanol and precision wipes. The available uATR device allows the operator to define the force used to push the sample onto the crystal. An optimal pressure improves the signal intensity, minimizes the noise, and provides better visualization of overlapping peaks. This force, displayed on the screen, is a dimensionless entity that ranges between 0 and 100. Analyzing entire tablets, given the more compact and tenacious nature of samples, it was not necessary to preliminarily investigate the most suitable force to be applied. On the other hand, with ground samples, this was deemed necessary. Consequently, as described above, the force applied was included as a factor of the DOE; eventually, it appeared that, in order to improve the intensity of the signals, the pressure had to be kept at 80.

### 3.3. Chemometric Methods

#### 3.3.1. ASCA

ANOVA-simultaneous component analysis (ASCA) [26] is a multivariate approach for the analysis of designed data, developed by combination of the analysis of variance (ANOVA) [31] and simultaneous component analysis (SCA) [32]. Considering a DOE based on three effects (α, β and γ), as the one investigated in the present study, ASCA starts with the ANOVA decomposition of the designed data matrix (X):

(1) $$X=X_{\alpha }+X_{\beta \;}+X_{\gamma \;}+X_{\alpha ,\beta \;}+X_{\alpha ,\gamma \;}+X_{\beta ,\gamma \;}+X_{\alpha ,\beta ,\gamma \;}+X_{E\;}$$

where$\;X_{\alpha }$, $X_{\beta \;},$ and $X_{\gamma \;}$are the matrices of the effects; $X_{\alpha ,\beta \;}$, $X_{\alpha ,\gamma \;}$, $X_{\beta ,\gamma \;}$, and $X_{\alpha ,\beta ,\gamma \;}$ take into account the interaction effects among factors and $X_{E\;}$contains unmodelled variability.

Afterward, SCA is calculated on each effect or interaction matrix. This approach, which is a multi-block extension of PCA to the case, provides a further decomposition of the variance among the diverse levels of each effect. Similar to PCA, the outcome of SCA can be inspected by means of score plot, which will allow the visualization of trends among samples belonging to different levels.

#### 3.3.2. SPORT-LDA

Sequential preprocessing through orthogonalization (SPORT) [33] is a signal preprocessing ensemble method applicable in a regression context, derived from the sequential and orthogonalized partial least squares (SO-PLS) multi-block approach [34,35], which exploits a sequential extraction of information from data blocks preprocessed by different pretreatments.

In the easiest case possible, i.e., the one where two different preprocessing approaches are tested on the dataset, called ***X*,** the data matrix and $X_{\mathrm{pret}\;}$the pre-processed blocks, the algorithm can be summarized as follows:

The response ***Y*** is fitted to the data block preprocessed by the first tested pretreatment ($X_{\mathrm{pret},1\;}$) by PLS.

***X*** is preprocessed by the second pretreatment (obtaining $X_{\mathrm{pret},2}$) and it is orthogonalized with respect to the scores extracted at step 1 (obtaining $X_{\mathrm{pret},2,\;\mathrm{orth}}$). This step ensures redundancies between blocks are removed [34,36].

The residuals from step 1 are fitted to $X_{\mathrm{pret},2,\;\mathrm{orth}}$.

The final regression model is calculated by summing up the contributions from steps 1 and 3:

(2) $$Y=X_{\mathrm{pret},1\;}B+X_{\mathrm{pret},2\;}C+E$$

where ***B*** and ***C*** are the regression coefficients and ***E*** the residuals. 

The SPORT method can be easily extended to the classification field, by combination with linear discriminant analysis [37]. This can be done by simply calculating LDA on the predicted ***Y*** in step 4.

## 4. Conclusions

The present work represents a feasibility study to realize an analytical method finalized to detect expired antibiotics tablets. The work focuses on a specific antibiotic drug and represents the preliminary study upstream of a larger-scale work.

In particular, the possibility of developing an analytical method both on intact and ground tablets was investigated; despite the possibility of fine tuning, the method on intact samples is preferable as it would allow a completely non-destructive determination.

In general, the work showed that it is possible, by combining ATR-FTIR and SPORT, to discriminate between expired and compliant tablets. The highest predictive accuracy (93.3% of correct classification rate in external validation, corresponding to 1 misclassified test sample over 15) was achieved by analyzing intact tablets. This outcome represents an excellent result because it gives indications regarding the possibility of determining the presence of expired drugs in a completely non-destructive way 

As stated above, several studies demonstrated the ability and sensitivity of infrared spectroscopy in quantifying Amoxicillin and/or Amoxicillin and Clavulanate Potassium tablets by performing destructive analysis on ground or uncoated samples [22,23]. Thus, it is not surprising that, after appropriate optimization, a qualitative model on ground tablets was able to distinguish, with sufficient accuracy, compliant and expired samples. Thus, making a mere comparison between destructive (ground samples) and non-destructive (whole tablets) methods and confirming the agreement of the classification models, it is plausible that the analysis of the intact tablets reflects the quality of the medicine in terms of concentration of the active ingredients. Although a quantitative analysis to confirm APIs’ concentration was not foreseen during conceptualization, it is highly unlikely that the expired samples would meet USP quality standards. Indeed, Augmentin formulation has been shown to be very sensitive to environmental conditions, such as temperature and humidity, as confirmed by the stability studies in the literature and the visual inspection of the analyzed expired brownish samples.

Clearly, this approach needs to be extended on a larger scale, expanding the number of samples, lots, and drugs investigated. In addition, it might be interesting to investigate to what extent an alteration in the coating materials, which dominate the spectra of whole tablets, may reflect an actual loss in quality (i.e., API’s concentration) of the drug by evaluating, in this case, the possibility of developing a quantitative non-destructive method. 

## Data Availability

Data is contained within the article.
